# Evaluating Methods of Preserving Aquatic Invertebrates for Microbiome Analysis

**DOI:** 10.3390/microorganisms10040811

**Published:** 2022-04-13

**Authors:** Stephanie N. Vaughn, Colin R. Jackson

**Affiliations:** Department of Biology, University of Mississippi, University, MS 38677, USA; snvaughn@go.olemiss.edu

**Keywords:** invertebrate, microbiome, sample preservation, crayfish, dragonfly

## Abstract

Research on the microbiomes of animals has increased substantially within the past decades. More recently, microbial analyses of aquatic invertebrates have become of increased interest. The storage method used while collecting aquatic invertebrates has not been standardized throughout the scientific community, and the effects of common storage methods on the microbial composition of the organism is unknown. Using crayfish and dragonfly nymphs collected from a natural pond and crayfish maintained in an aquarium, the effects of two common storage methods, preserving in 95% ethanol and freezing at −20 °C, on the invertebrate bacterial microbiome was evaluated. We found that the bacterial community was conserved for two sample types (gut and exoskeleton) of field-collected crayfish stored either in ethanol or frozen, as was the gut microbiome of aquarium crayfish. However, there were significant differences between the bacterial communities found on the exoskeleton of aquarium crayfish stored in ethanol compared to those that were frozen. Dragonfly nymphs showed significant differences in gut microbial composition between species, but the microbiome was conserved between storage methods. These results demonstrate that preserving field-collected specimens of aquatic invertebrates in 95% ethanol is likely to be a simple and effective sample preservation method for subsequent gut microbiome analysis but is less reliable for the external microbiome.

## 1. Introduction

Over the past twenty years, the human microbiome has been at the forefront of health-related research [1]. This has largely been because of an increase in technologies allowing for next generation 16S rRNA gene sequencing, and various human diseases are now known to be the result of gut dysbiosis [2,3,4]. Advances in more efficient DNA sequencing methods, such as next generation sequencing and Illumina technology, have enabled scientists to pursue microbiome research beyond that of humans [2,5,6,7]. Substantial interest over the past decade has focused on host-related microbiomes of other animals. However, throughout this increase in animal microbiome studies ranging from humans to invertebrates, there have been inconsistencies between findings, partly because of differences in sample storage methods [4,5,8,9,10,11,12]. This is especially pronounced in aquatic invertebrates where field collection of samples often requires immediate storage, yet the most suitable method for conserving the microbiome of samples has not been defined and has seldom been investigated [13,14,15].

Preserving aquatic invertebrate samples is crucial to accurately analyzing the bacterial community associated with the specimens of interest. Many studies have incorporated some method of sample preservation prior to later analyses, yet the impact of storage methods on the microbial community of these samples is poorly understood [8,13,14,16,17]. This is important for samples collected in the field, where there may be substantial travel time between the sampling site and laboratory. Common forms of sample preservation for field-collected invertebrates include flash freezing with liquid nitrogen, freezing, and storage in ethanol or RNAlater [13,14,16]. When the goal of the storage is to preserve the invertebrate specimen itself, these methods may be sufficient, but when wanting to conserve the bacterial community associated with these specimens, the impact of these methods are not well understood. While a storage method must preserve the microbiome of a particular sample, there are also logistical considerations, especially in the context of fieldwork, and approaches differ in their availability, ease of use in the field, and cost.

The aim of this study was to determine the effects that two common storage methods, preserving samples in ethanol or freezing, have on the microbiome aquatic invertebrates. Aquatic invertebrates are of increasing interest for microbiome studies because of their significant roles in marine and freshwater ecosystems [18,19]. Ecosystem services provided by aquatic invertebrates include bioturbation, filter feeding, nutrient and chemical retention, and food web interactions [18,20]. Here, we determine how storing samples in 95% ethanol and freezing at −20 °C affected the bacterial composition of gut and exoskeleton samples from one species of crayfish (*Procambarus vioscai paynei*) and three species of dragonfly nymphs (*Libellula luctuosa*, *Pachydiplax longipennis*, and *Erythemis simplicicollis*) collected from a natural pond, as well as a second species of crayfish *(Faxonius virilis*) maintained in an aquarium to help standardize their microbiome prior to collection. Partial 16S rRNA gene sequences obtained from high throughput sequencing were classified into amplicon sequence variants (ASVs) to assess bacterial microbiome composition and alpha diversity of each specimen, and beta diversity between specimens. We show that preservation in 95% ethanol, as is commonly used to preserve invertebrate specimens for other purposes, is a valid method for the preservation of gut microbiomes of aquatic invertebrates, and potentially suitable for the preservation of the external, exoskeleton microbiome.

## 2. Materials and Methods

### 2.1. Specimen Collection and Processing

Multiple experiments were conducted to assess the effects of storage method on the microbiomes of aquatic invertebrates. The first experiment used field-collected aquatic invertebrates: ten crayfish (*Procambarus vioscai paynei*) and 18 dragonfly nymphs (six each of *Libellula luctuosa*, *Pachydiplax longipennis*, *Erythemis simplicicollis*). All organisms were collected on 5/5/2021 from ponds at the University of Mississippi Field Station (UMFS; Lafayette County, MS, USA). Numbers of each species of invertebrate were determined from what was caught. Immediately after collection, specimens were placed into buckets of pond water and transported (1 h) to the laboratory at University of Mississippi main campus. At the laboratory, five crayfish were placed, individually, into 95% ethanol while five were sealed, individually, in sterile bags and frozen in a −20 °C freezer. Similarly, three dragonfly nymphs of each species were placed, individually, in 95% ethanol and three were placed in sterile bags in a −20 °C freezer. Specimens were preserved for almost three months (83 days) before being sampled for microbiome composition.

In a second experiment, a group of commercially acquired crayfish *(Faxonius virilis*) were housed in a 30-gallon aquarium in the laboratory for 24 days in an attempt to reduce individual to individual variation in their microbiome. Aquarium crayfish were fed a standardized diet of commercial food pellets (Hikari Crab Cuisine, Kyorin Co., Ltd., Himeji City, Japan), and Pro PlecoWafers, Tetra, Melle, Germany). After 24 d, 14 visibly healthy crayfish were removed and seven were placed, individually, into 95% ethanol while the other seven were sealed in sterile bags and frozen at −20 °C, as per the field-collected invertebrates. Specimens from the aquarium experiment were preserved for two months (60 days) before being dissected.

For all crayfish, exoskeleton samples were collected by gently rinsing each crayfish quickly in sterile water to remove non-attached microorganisms. This rinsing also served to partly thaw frozen specimens and removed residual ethanol from ethanol-preserved samples. Samples were then scrubbed gently three times for 30 s each using a sterile toothbrush. Material that was scrubbed off was placed into the initial buffer solution (CD1) from a PowerSoil Pro kit (Qiagen, Germantown, MD, USA). Following exoskeleton scraping, crayfish were dissected by making an incision on the dorsal side of the telson and up the abdomen and the gut extracted. The extracted gut samples were placed directly into bead beating tubes containing buffer solution (CD1) from the PowerSoil Pro kit. Dragonfly nymphs were too small to assess for exoskeleton microbiome composition so only the gut microbiome was examined. The guts of dragonfly nymphs were obtained by cutting through the dorsal portion of the abdominal segments and placing the gut into bead beating tubes containing buffer solution (CD1) from PowerSoil Pro kit.

### 2.2. DNA Extraction, Amplification, and Sequencing

DNA was extracted from all sample types using the PowerSoil Pro kit and following manufacturer’s instructions. A 250 bp portion of the V4 region of the bacterial 16S rRNA gene in each sample was sequenced using a dual-index 8-nucleotide barcoding approach [21]. This approach uses a single round of PCR, reducing the risk of amplification artifacts. Following amplification, the presence of amplicons was verified using agarose gels, amplification products standardized using SequalPrep plates (Life Technologies, Grand Island, NY, USA), and barcoded products pooled prior to sequencing. The assembled library was spiked with 20% PhiX [22,23] and sequenced on an Illumina MiSeq at the University of Mississippi Medical Center (UMMC) Molecular and Genomics Core Facility.

Raw sequence files (fastq) were processed using the standard 16S rRNA pipeline of the DADA2 package version 1.12.1 [24] within R version 1.3.1073 [25]. At least 80% of sequences from each sample were retained following quality trimming: truncLen = c(240,160), maxN = 0, maxEE = c(2,2), truncQ = 2. Quality profile plots were inspected to ensure proper quality of trimmed reads. During merging of reads, sequences were trimmed further to account for any overhang (trimOverhang = TRUE) and sequences shorter than 250 base pairs (bp) and longer than 256 bp were trimmed. Chimeras were removed using the “consensus” method. Sequences were classified against the RDP v.18 database [26]. Final amplicon sequence variant (ASV) data was transformed into relative abundance (% sequence reads) of microbial taxa for further compositional analysis.

### 2.3. Statistical Analyses

Alpha diversity was assessed using the Inverse Simpson’s Index to measure overall bacterial species diversity and Observed Species Richness (richness based on repeated subsampling of the rarefied number of sequences) to determine richness of ASVs. Two-way analysis of variance (ANOVA) tests were performed on samples to determine differences in mean diversity and richness between storage method (frozen or ethanol) and sample type (gut or exoskeleton) for crayfish, or storage method and species for dragonfly nymphs. One-way ANOVAs were performed to further asses the differences in evenness and richness estimates based on crayfish separated by their storage method and corresponding sample type (gut, exoskeleton). Effect sizes were calculated using the pwr package of R to assess statistical importance of ANOVA results. No *a priori* hypothesis were stated, therefore, TukeyHSD post hoc tests were performed to further assess the differences among group means of significant variables. Multivariate analysis of variance (MANOVA) tests were used to assess if bacterial phyla differed between storage method for each invertebrate/experiment (aquarium crayfish, pond crayfish, and dragonfly nymphs). Bray–Curtis dissimilarity matrices compared structural differences of bacterial communities by storage method, and sample type for crayfish, or species for dragonflies. Permutational multivariate analysis (PERMANOVA) tests using Bray–Curtis distance matrices were performed to determine whether storage method, sample type, and/or species significantly affected the composition of the microbiome. Non-metric multidimensional scaling (NMDS) ordinations were created using the metaMDS function in the Vegan package [27] of R to visualize these differences. The most frequent ASVs in ethanol-preserved and frozen gut and exoskeletons of crayfish samples was determined using the “microbiome” package version 1.12.0 [28] in R where “core” AVSs were specified as those most commonly found in samples of each category.

## 3. Results

### 3.1. Sequence Counts

Initial DADA2 analysis yielded 3810 ASVs from a total of 815,362 16S rRNA sequence reads of the V4 region. Following trimming, merging, chimera removal, and classification against RDP (version 18), 3693 ASVs from 671,032 sequences were retained for the full dataset. Independent *t*-tests were run to determine any potential effect that storage method may have on the amount of sequence reads retained per sample. Aquarium crayfish showed a significantly higher number of sequence reads for gut samples from ethanol-preserved crayfish (15,057 ± 7782 sequences) compared to those from frozen crayfish (7227 ± 3735; *p* < 0.01, t(13) = −2.374). Exoskeleton samples from frozen field-collected crayfish showed a significantly higher number of reads compared to exoskeleton samples from ethanol-preserved field-collected crayfish (20,889 ± 2678 and 7403 ± 2861, respectively; *p* < 0.001, t(7) = 11.41). Rarefaction parameters were set to retain samples containing more than 2000 sequences for crayfish, which subsequently removed four samples: one frozen aquarium crayfish gut sample, two field-collected ethanol-preserved crayfish gut samples, and one field-collected ethanol-preserved crayfish exoskeleton samples. Dragonfly nymphs showed lower overall numbers of sequence reads retained compared to that of crayfish. Thus, rarefaction parameters for dragonfly nymph samples were set to 1000 sequences which subsequently removed six dragonfly nymphs. Dragonfly nymph samples showed no significant difference between the number of sequence reads retained in ethanol-preserved compared to that of frozen samples.

### 3.2. Differences in the Crayfish Microbiome between Sample Types and Preservation Method

**Figure 1:**
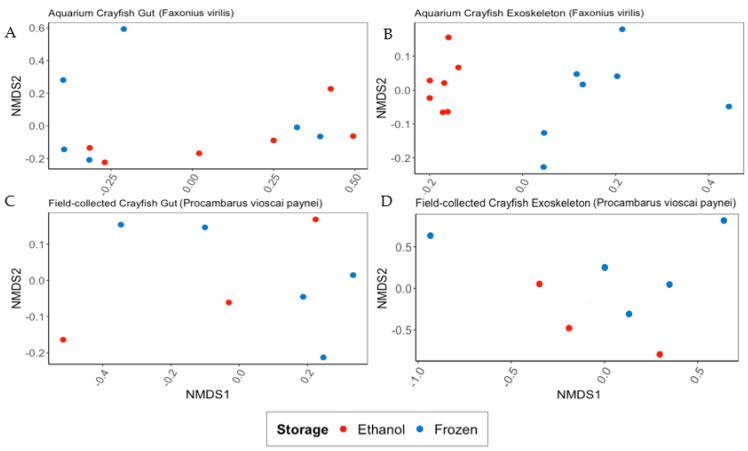
NMDS ordinations based on Bray–Curtis dissimilarity scores for bacterial communities of aquarium (*Faxonius virilis*; (**A**,**B**)) and field-collected (*Procambarus vioscai paynei*; (**C**,**D**)) crayfish based on sample preservation method (95% ethanol or frozen at −20 °C) and separated by sample type (gut, (**A**,**C**), or exoskeleton, (**B**,**D**)). Sample preservation method within each plot is represented by color. Gut and exoskeleton communities were significantly different for both aquarium crayfish (*p* < 0.001, F = 11.554) and field-collected crayfish (*p* < 0.05, F = 3.3). Sample preservation method only produced a significant difference in the bacterial community for exoskeleton samples from aquarium crayfish (*p* < 0.01, F = 4.837; (**B**)).

There were significant differences in overall microbiome composition between gut and exoskeleton samples for both aquarium (*F. virilis*) and field-collected crayfish (*P. vioscai paynei*; Adonis PERMANOVA analyses based on Bray–Curtis distances showed *p* < 0.001, F = 11.554 and *p* < 0.021, F = 3.348, respectively). The gut microbiome of aquarium crayfish showed no significant difference in overall bacterial composition based on storage method (ethanol or frozen; Figure 1A); however, there was a significant difference in overall bacterial community composition between the ethanol-preserved and frozen exoskeleton samples of aquarium crayfish (*p* < 0.01, F = 4.837; Figure 1B). Neither gut nor exoskeleton microbiomes of field-collected crayfish differed in terms of overall bacterial composition when comparing storage method (Figure 1C,D).

**Figure 2:**
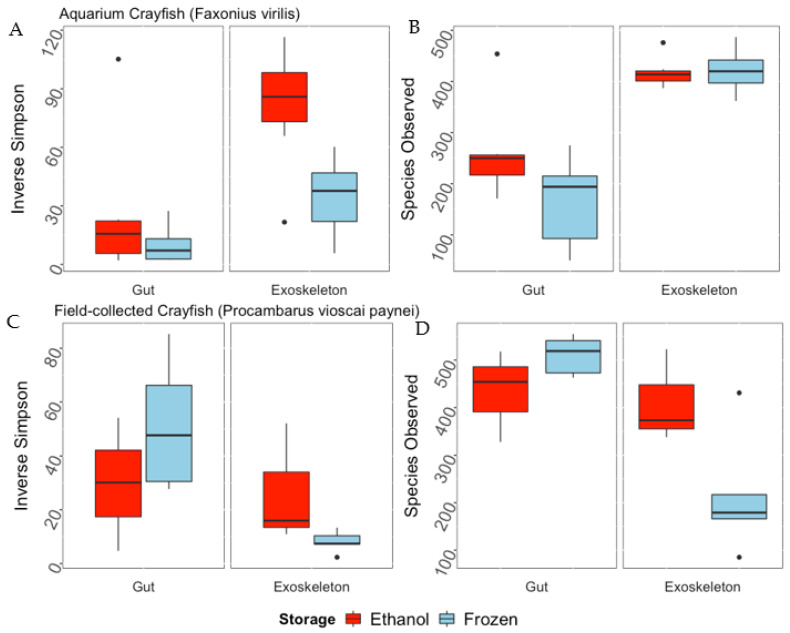
Alpha diversity metrics (Inverse Simpson’s Index, (**A**,**C**); Observed Species Richness, (**B**,**D**)) derived from gut or exoskeleton bacterial communities of aquarium-maintained (*Faxonius virilis*; (**A**,**B**)) and field-collected (*Procambarus vioscai paynei*; (**C**,**D**)) crayfish collected and stored under different conditions. Samples are separated into their corresponding storage method (95% ethanol, frozen). Boxes show the interquartile range/distribution of values measured in each metric with the black solid line representing the median value from sample type. Vertical lines represent the highest and lowest values associated with each sample type. Dots represent outliers from each group. Observed Species Richness was significantly different between exoskeleton and gut samples for aquarium and field-collected crayfish (*p* < 0.001, F = 56.312 and *p* < 0.01, F = 15.874, respectively), as was the Inverse Simpson’s Index (*p* < 0.01, F = 13.522 for aquarium and *p* < 0.05, F = 8.246 for field-collected). Sample preservation method was only significant for the Inverse Simpson’s Index of exoskeleton samples from aquarium crayfish (*p* < 0.01, F = 11.537; (**B**)).

There was a significant difference in the Inverse Simpson’s Index and Observed Species Richness based on microbiome location for the aquarium-maintained *F. virilis*, with the exoskeleton microbiome being richer (*p* < 0.001, F = 56.312) and more diverse (*p* < 0.01, F = 13.522) than the gut microbiome. This was particularly pronounced for Species Observed, where exoskeleton samples predicted approximately 400 observed bacterial species compared to 150–300 in the gut community (Figure 2A). The Inverse Simpson’s Index was significantly higher in exoskeleton microbiomes of ethanol-preserved of *F. virilis* compared to those from frozen crayfish (*p* < 0.01, F = 11.537; Figure 2B), although storage method did not affect the species diversity of gut microbiomes for these samples (Figure 2B). Field-collected *P. vioscai paynei* showed significant differences in Observed Species Richness and the Inverse Simpson’s Index between gut and exoskeleton samples, with gut microbiomes being higher for both indices (*p* < 0.01, F = 15.87 and *p* < 0.05, F = 8.246, respectively). Neither gut nor exoskeleton samples of field-collected crayfish showed significant differences in diversity indices based on sample storage method (Figure 2C,D). Cohen’s effect size was medium to large (0.33–0.91) for all comparisons between frozen and ethanol-preserved samples, with the exception of aquarium-maintained *F. virilis* (0.06).

**Figure 3:**
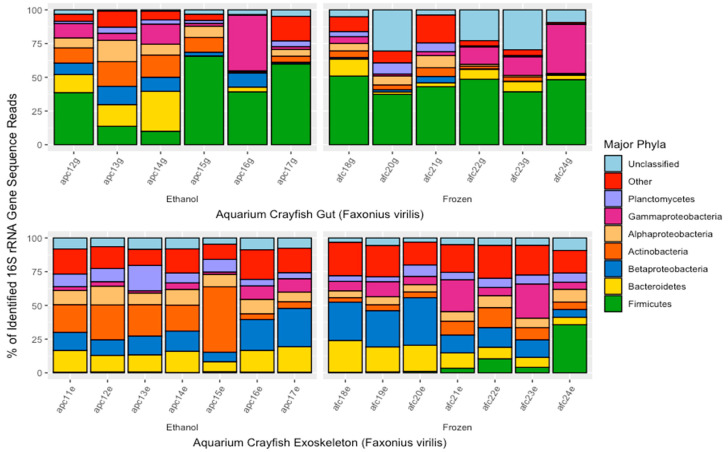
Major bacterial phyla found in the gut and exoskeleton microbiomes of aquarium-maintained crayfish (*Faxonius virilis*) as determined from percent of 16S rRNA gene sequence reads. Each bar represents one individual and are separated by sample type (gut or exoskeleton) and sample storage method (in 95% ethanol or frozen at −20 °C). Sample names are located on the *x*-axis and correspond to the location (i.e., a = aquarium), storage method (i.e., *p* = ethanol-preserved, f = frozen), the number order of crayfish collection, storage, and subsequent dissection, and the sample type being analyzed (i.e., g = gut, e = exoskeleton).

There were significant differences in the major bacterial phyla found in gut and exoskeleton samples of aquarium *F. virilis* crayfish (MANOVA; *p* < 0.01, F = 11.554; Figure 3). Based on the proportions of 16S rRNA gene sequences, major bacterial phyla (or subphyla of Proteobacteria) found in the guts of *F. virilis* were the Firmicutes (35.6% of sequences), Bacteroidetes (12.0%), Actinobacteria (10.3%), Gammaproteobacteria (9.50%), Alphaproteobacteria (9.10%), Betaproteobacteria (8.58%), and Planctomycetes (4.35%). Major bacterial phyla/subphyla in exoskeleton samples of aquarium-maintained *F. virilis* were the Bacteroidetes (20.3%), Betaproteobacteria (16.4%), Actinobacteria (15.0%), Alphaproteobacteria (13.9%), Planctomycetes (9.01%), Verrucomicrobia (3.51%), and Deltaproteobacteria (3.19%). Bacterial phyla that differed significantly in their representation between gut and exoskeleton samples were the Firmicutes (MANOVA; *p* < 0.001, F = 19.154) which were proportionally more abundant in gut samples (35.0% more) and Alphaproteobacteria (*p* < 0.05, F = 7.168) which were proportionally more abundant in exoskeleton samples (4.8% more). While there was some variability in the proportions of major bacterial phyla in the gut microbiomes of *F. virilis* between ethanol-preserved and frozen samples, none of this variability was significant (MANOVA; *p* > 0.05). The exoskeleton microbiomes of aquarium crayfish did show differences in the composition of major bacterial phyla based on sample storage method, with the percentage representation of Betaproteobacteria (MANOVA; *p* < 0.001, F = 2.812), and Bacteroidetes (*p* < 0.001, F = 26.264), being significantly higher in ethanol-preserved samples (+8.19% and +10.7%, respectively) and the percentage of Actinobacteria being +16.2% higher in frozen samples (*p* < 0.01, F = 11.522).

**Figure 4:**
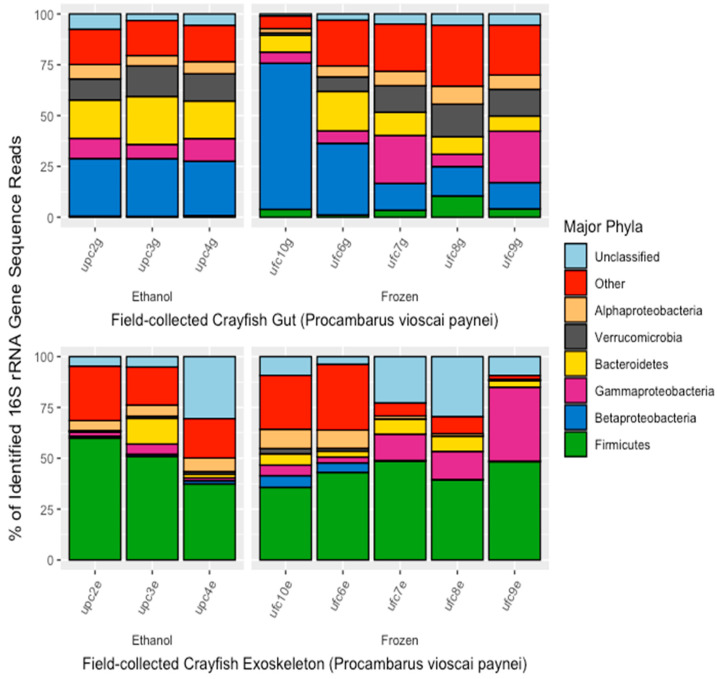
Major bacterial phyla found in the gut and exoskeleton microbiomes of field-collected crayfish (*Procambarus vioscai paynei*) as determined from percent of 16S rRNA gene sequence reads. Each bar represents one individual and are separated by sample type (gut or exoskeleton) and sample storage method (in 95% ethanol or frozen at −20 °C). Sample names are located on the *x*-axis and correspond to the location (i.e., a = aquarium), storage method (i.e., *p* = ethanol-preserved, f = frozen), the number order of crayfish collection, storage, and subsequent dissection, and the sample type being analyzed (i.e., g = gut, e = exoskeleton).

As with aquarium crayfish, the major bacterial phyla/subphyla in the microbiomes of field-collected *P. vioscai paynei* crayfish were significantly different between exoskeleton and gut samples (MANOVA; *p* < 0.05, F = 3.48; Figure 4). The gut microbiome (Figure 4) was primarily composed of Firmicutes (49.4% of sequences), Cyanobacteria (6.12%), Alphaproteobacteria (5.72%), Planctomycetes (5.47%), Bacteroidetes (4.87%%), and Actinobacteria (4.45%). The major bacterial phyla making up the exoskeleton microbiome were Betaproteobacteria (22.8%), Bacteroidetes (15.5%), Verrucomicrobia (12.5%), Gammaproteobacteria (12.4%), Alphaproteobacteria (7.14%), Actinobacteria (6.98%), and Planctomycetes (5.61%). Gut and exoskeleton samples from field-collected crayfish differed in their percentage representation of Actinobacteria (MANOVA; *p* < 0.05, F = 5.135, +2.53% in exoskeleton) and Verrucomicrobia (*p* < 0.01, F = 11.280, +11.53% in exoskeleton). Storage method had no significant effect on proportions of any of the major bacterial phyla/subphlya in the gut or exoskeleton microbiome for field-collected crayfish.

### 3.3. Dominant ASVs by Sample Type and Preservation Method

The most frequently observed ASVs from aquarium and field-collected crayfish of each sample type preserved in ethanol of frozen were determined and classified by their finest identified taxonomic level. For gut microbiome samples from aquarium crayfish (*F. virilis*), four of the six most abundant ASVs were the same regardless of the method of sample preservation (Table 1). Those that were not specifically identified as the same ASV all classified within the Proteobacteria phylum (ASV34, ASV69, ASV25, and ASV27). ASV1, ASV4, and ASV9 were the three most abundant ASVs within both frozen and ethanol-preserved gut samples; however, the most abundant in these samples, ASV1, could not be identified further than the phylum level (Firmicutes). Consistency in dominant ASVs between sample storage procedures was much less for the exoskeleton samples from aquarium crayfish, with only one of the six most frequent ASVs being in the core microbiome of both ethanol-preserved and frozen samples (ASV9, identified as a member of *Mycobacterium*).

The most frequently detected ASVs in the gut microbiome of field-collected *P. vioscai paynei* were generally the same regardless of sample storage method, with five of the six most common ASVs being found in both ethanol-preserved and frozen gut samples (Table 2). Sample storage method had a greater impact on the exoskeleton microbiome of field-collected crayfish, with only two of six common ASVs (ASV12 identified as *Sphaerotilus*, and ASV 16 identified as *Verrucomicrobium*) being the same for ethanol-preserved and frozen samples (Table 2).

### 3.4. Patterns in the Dragonfly Nymph Microbiome by Species and Preservation Method

**Figure 5:**
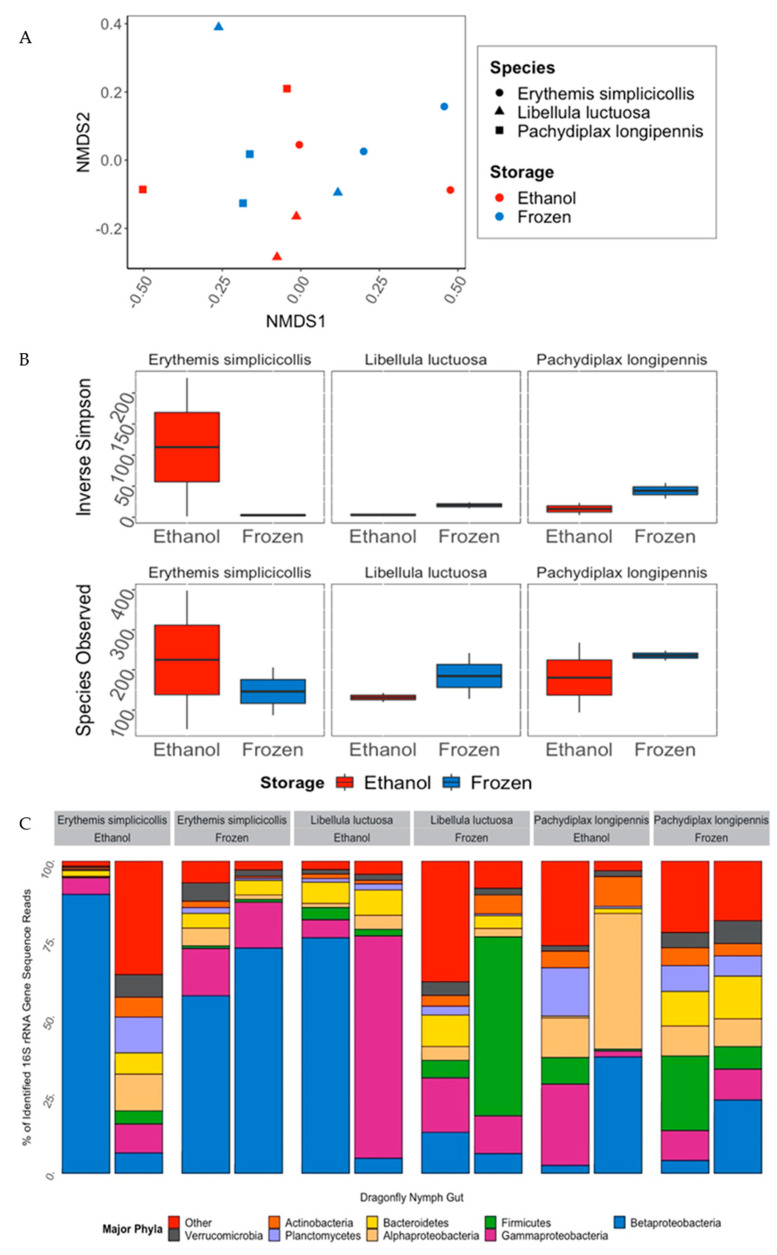
Diversity patterns in gut microbiome of three species of dragonfly nymphs (*E. simplicicollis, L. luctuosa,* and *P. longipennis)* that were preserved in 95% ethanol or frozen at −20 °C. (**A**) NMDS ordination based on Bray–Curtis dissimilarity scores (**B**) Alpha diversity plots of Inverse Simpson’s Index and Observed Species Richness separated by host species and preservation method. There were no significant differences in diversity indices between preservation methods for any species. (**C**) Major bacterial phyla found in the gut of dragonfly nymphs as determined from percent of 16S rRNA gene sequence reads. Each bar represents one individual and are separated by storage method (gut or exoskeleton) and nymph species.

Gut microbiomes of the three species of dragonfly nymphs (*E. simplicicollis*, *L. luctuosa*, *P. longipennis*) were significantly different from each other based on species (Adonis PERMANOVA analyses based on Bray–Curtis distances; *p* < 0.05, F = 1.844; Figure 5A). There was, however, no difference in overall microbiome composition based on sample preservation method (Figure 5A). Similarly, there were no significant differences in the alpha diversity indices (Inverse Simpson’s Index, Observed Species Richness) of dragonfly gut microbiomes based on sample preservation method or, for that matter, by host species (Figure 5B). Dominant bacterial phyla (subphyla for Proteobacteria) in the 16S rRNA gene sequence dataset recovered from dragonfly nymphs were the Betaproteobacteria (32.7% of recovered sequences), Gammaproteobacteria (16.6%), Firmicutes (9.61%), Alphaproteobacteria (8.90%), Bacteroidetes (6.18%), and Planctomycetes (4.35%) (Figure 5C). The only phyla that showed a significant difference in relative abundance based on sample storage method, were the Bacteroidetes (MANOVA; *p* < 0.05, F = 7.242), which were found at a higher proportion in the frozen *P. longipennis* samples (22.6% more abundant) compared to ethanol-preserved samples of the same species.

## 4. Discussion

While the number of studies analyzing the host-associated bacterial communities of aquatic invertebrates is increasing, there are few studies analyzing the effects that preservation has on stored specimen’s microbiome. Of the few studies previously analyzing the effects that preservation has on any microbiome sample [8,16,17,29], they have primarily focused on preserving fecal specimens of vertebrates rather than preserving the entire host as we did for the aquatic invertebrates sampled in this study. Furthermore, the results of the previous studies were inconclusive as to which storage method would be ideal for microbiome preservation of their samples, leaving the decision to the investigator. However, given that ethanol is one of the most commonly used preservation methods for storing aquatic invertebrates [30,31,32,33], it is critical to understand the effects ethanol has on the bacterial community of host species before choosing and standardizing field-preservation methods or analyzing invertebrates stored for the long-term in collections.

Consistent in all analyses were the differences between the gut and exoskeleton microbiomes of both crayfish species and the differences between species for dragonfly nymphs. In the current study, these differences were apparent regardless of sample storage method (freezing, preservation in 95% ethanol) suggesting that broad ecological patterns are likely to be detected regardless of how samples are preserved. The bacterial communities associated with aquatic macroinvertebrates (e.g., crayfish) has often been found to differ based on the locality of the sample [34,35,36,37]. Skelton et al. [36] characterized the carapace and gill microbiomes of the crayfish species, *Cambarus sciotensis*, the first characterization of any crayfish microbiome to their knowledge. They found that the bacterial community of the exoskeleton was largely influenced by the water column that crayfish were collected from [36]. That study, along with more recent studies [34,35,37], and the results of the current study show the differences in bacterial diversity and major bacterial taxa between different parts of the crayfish body, and suggest that each area may have its own functional role for the well-being of the host.

When investigating multiple insect species (*Pieris rapae* (Lepidoptera), *Arphia conspersa* (Orthoptera), *Epilachna varivestis* (Coleoptera), *Apis mellifera* (Hymenoptera)) preserved by various methods, Hammer et al. [15] found similar results to our study, in that they were able to distinguish the microbiomes between different species, regardless of storage method [13]. However, they declared that no single storage method had a significantly greater preservation effect on the bacterial community of the insects than any other and suggest that storage method be determined by the investigator based on cost and efficiency (i.e., travel time from field to laboratory). Along with our findings that 95% ethanol was a suitable sample preservation method for microbiome analyses of crayfish and other aquatic invertebrates, this suggests the potential that samples that have been stored long-term in ethanol, as is common in collections, could be characterized to assess their microbiomes. That said, assessing the effects of longer-term storage in ethanol should be a priority, although such studies would, by nature, take a much longer period of time.

The most dominant taxa in the gut microbiome of aquarium crayfish, both ethanol-preserved and frozen, were Firmicutes, consistent with previous studies analyzing gut bacteria of crayfish [34,35]. Exoskeleton samples from these same crayfish showed the greatest differences in microbiome composition based on preservation method, with ethanol-preserved vs. frozen individuals differing in terms of dominant phyla, major ASVs, and alpha diversity indices. Looking at the differences, there is the possibility that ethanol-preservation decreased the percentages of dominant taxa making the exoskeleton bacterial community more even, although it is equally possible that freezing may have had the opposite effect. Sampling the microbiome from crayfish immediately after collection would be useful as a control for direct comparisons to preserved samples, but it is generally necessary to freeze crayfish prior to scrubbing the exoskeleton, and the humane way of euthanizing invertebrates typically entails freezing or ethanol immersion, making microbiome sampling from freshly collected individuals difficult.

Gut microbiomes from the field-collected crayfish *P. vioscai paynei* were similar between ethanol-preserved and frozen samples with Betaproteobacteria being the most prevalent phyla identified. This similarity in microbiome composition regardless of preservation method was further supported through alpha and beta diversity indices. Although Betaproteobacteria accounted for the greatest percentage of sequences in these samples, the most frequently detected ASVs in the gut microbiomes of both ethanol-preserved and frozen *P. vioscai paynei* were identified as belonging to Firmicutes phylum, taxa that have been regarded as common in the guts of other crayfish species [34,35]. Firmicutes were the most prevalent phylum in exoskeleton samples of the field-collected crayfish, regardless of preservation method, although the most commonly detected ASVs were identified as members of the phyla Verrucomicrobia and Proteobacteria. While there was variability in the most frequently identified ASVs in the exoskeleton microbiome of ethanol-preserved and frozen samples, alpha and beta diversity metrics suggested that preservation method had little impact on the overall microbiome associated with the exoskeleton of field-collected crayfish.

The gut microbiomes of all three species of dragonfly nymphs were dominated by 16S rRNA gene sequences classified within phylum Proteobacteria, which is consistent with previous studies analyzing the gut microbiome of dragonfly nymphs [38,39]. Those previous studies also found that host species had a significant effect on the gut microbial community of dragonflies, and the three species of nymphs examined in this study (*Libellula luctuosa*, *Pachydiplax longipennis*, *Erythemis simplicicollis*) were also found previously to have distinct gut microbiomes [38]. Preservation method had no effect on any of the microbiome community parameters that we examined, suggesting that future studies could be conducted to look at the gut microbiomes of dragonfly nymphs, as well as other aquatic insects, that are commonly stored in ethanol. That said, larger studies on the effects of sample preservation on the aquatic insect microbiome are needed, as the results of this portion of our study are potentially limited by a relatively low sample size.

Using a consistent method of sample preservation within a study is important to accurately assess ecological patterns in microbiome composition. This is evident from our finding that, while most types of samples yielded similar microbiome data regardless of whether samples were frozen or preserved in ethanol, the exoskeleton microbiome of *F. virilis* differed substantially with preservation method. Others have found significant differences between frozen and ethanol-preserved tadpole feces (*Nanorana parkeri*), although that was acknowledged, in part, as being due to thawing of frozen samples during transport to the laboratory [8]. Of the few studies that have analyzed the effect of perseveration method on the microbiome of other aquatic invertebrates, most have concluded that the microbiome of organisms is capable of being retained after specimen storage [13,14,15]. From the current study, it was determined that 95% ethanol is an acceptable method to conserve the internal microbiome and a potential way to conserve the external microbiome of aquatic invertebrates. The potential for ethanol to be used as quick and economical method of preserving specimens in the field shows promise and would reduce potential issues with the transportation of frozen specimens for later microbiome analysis.

Standardized protocols for preserving aquatic invertebrate samples gives the scientific community the opportunity to directly compare the effects of species, habitat, climate, nutrients, etc., on the microbiome of these aquatic organisms. Ethanol is one of the most frequently used preservation methods for storage of aquatic invertebrate specimens for study and in museum collections, because of its ability to fix specimen, morphologically and molecularly [30,31,32,33], and our study shows that it can also be used for preservation of the gut microbiome. One limitation of our study, however, could be the length of time that samples were stored (almost three months) and future work could examine how longer storage times relate to the reliability of recovering a representative microbiome community, especially if long-term ethanol-preserved specimens, such as in museum collections [30,31,32,33], are to be examined. Regardless, this initial study shows that ethanol-preservation was as successful as freezing in conserving the gut microbial community of a variety of aquatic invertebrates. Future work should further examine the impacts of sample preservation methods on the microbiome of other aquatic animals that are commonly preserved in ethanol, such as mollusks and even vertebrates.

## Figures and Tables

**Table 1:** The core microbiome (most frequently identified ASVs from each sample) of aquarium crayfish (*Faxonius virilis*) gut and exoskeleton samples, separated into those preserved in 95% ethanol or frozen at −20 °C.

Aquarium Crayfish	ASV	Identification	Frequency ^a^	Relative Abundance ^b^	CI (+/−)
Gut Ethanol	ASV 1	Firmicutes (Firmicutes)	6/6	24.2%	8.39%
	ASV 4	*Flavobacterium* (Bacteroidetes)	6/6	7.10%	2.55%
	ASV 9	*Mycobacterium* (Actinobacteria)	6/6	3.67%	1.22%
	ASV 34	*Gemmobacter* (Alpharoteobacteria)	6/6	2.32%	0.81%
	ASV 33	*Mycobacterium* (Actinobacteria)	6/6	1.77%	0.60%
	ASV 69	*Dechloromonas* (Betaproteobacteria)	5/6	1.28%	0.44%
Gut Frozen	ASV 1	Firmicutes (Firmicutes)	6/6	21.8%	5.01%
	ASV 4	*Flavobacterium* (Bacteroidetes)	6/6	5.58%	1.07%
	ASV 9	*Mycobacterium* (Actinobacteria)	6/6	5.23%	0.63%
	ASV 27	*Hydromonas* (Betaproteobacteria)	6/6	3.93%	1.48%
	ASV 25	*Citrobacter* (Gammaproteobacteria)	5/6	2.10%	0.12%
	ASV 33	*Mycobacterium* (Actinobacteria)	6/6	1.82%	0.25%
Exoskeleton Ethanol	ASV 3	Kineosporiaceae (Actinobacteria)	7/7	15.5%	1.93%
	ASV 31	Bacteroidetes (Bacteroidetes)	7/7	2.14%	0.18%
	ASV 19	Phycisphaeraceae (Planctomycetes)	6/7	1.98%	0.60%
	ASV 21	Pirellulaceae (Planctomycetes)	7/7	1.84%	0.13%
	ASV 28	*Fimbriiglobus* (Planctomycetes)	6/7	1.26%	0.07%
	ASV 9	*Mycobacterium* (Actinobacteria)	7/7	1.11%	0.10%
Exoskeleton Frozen	ASV 1	Firmicutes (Firmicutes)	7/7	19.1%	4.04%
	ASV 4	*Flavobacterium* (Bacteroidetes)	7/7	4.81%	0.89%
	ASV 9	*Mycobacterium* (Actinobacteria)	7/7	4.56%	0.55%
	ASV 27	*Hydromonas* (Betaproteobacteria)	5/7	3.36%	1.18%
	ASV 25	*Citrobacter* (Gammaproteobacteria)	6/7	1.81%	0.24%
	ASV 33	*Mycobacterium* (Actinobacteria)	7/7	1.56%	0.22%

^a^ Frequency was determined from the number of individuals found with that ASV. ^b^ Relative abundance was determined from the total number of each ASV identified within each storage group (i.e., ethanol and frozen).

**Table 2:** The core microbiome (most frequently identified ASVs from each sample) of field-collected crayfish (*Procambarus vioscai paynei*) gut and exoskeleton samples, separated into those preserved in ethanol or frozen at −20 °C.

Field-Collected Crayfish	ASV	Identification	Frequency ^a^	Relative Abundance ^b^	CI (+/−)
Gut Ethanol	ASV 7	*Catenococcus* (Gammaproteobacteria)	3/3	16.6%	2.96%
	ASV 1	*Rhodobacter* (Firmicutes)	3/3	12.7%	3.22%
	ASV 15	Bacilli (Firmicutes)	3/3	10.3%	1.86%
	ASV 11	*Clostridium_XlVb* (Firmicutes)	3/3	7.72%	1.81%
	ASV 22	Firmicutes (Fimicutes)	3/3	5.73%	1.65%
	ASV 32	*Dysgonomonas* (Bacteroidetes)	3/3	4.73%	0.75%
Gut Frozen	ASV 17	Firmicutes (Firmicutes)	5/5	14.5%	3.51%
	ASV 1	Firmicutes (Firmicutes)	5/5	12.5%	1.53%
	ASV 22	Firmicutes (Firmicutes)	3/5	4.77%	1.03%
	ASV 11	*Clostridium_XlVb* (Firmicutes)	5/5	4.53%	0.86%
	ASV 32	*Dysgonomonas* (Bacteroidetes)	3/5	3.41%	1.01%
	ASV 15	Bacilli (Firmicutes)	3/5	2.09%	0.39%
Exoskeleton Ethanol	ASV 83	Methylococcaceae (Gammaproteobacteria)	3/3	3.78%	1.44%
	ASV 16	*Verrucomicrobium *(Verrucomicrobia)	3/3	3.37%	1.24%
	ASV 68	Kineosporiaceae (Actinobacteria)	3/3	3.32%	0.83%
	ASV 115	Verrucomicrobiaceae (Verrucomicrobia)	3/3	2.73%	0.54%
	ASV 171	Verrucomicrobia (Verrucomicrobia)	3/3	1.62%	0.13%
	ASV 193	Micrococcales (Actinobacteria)	3/3	1.05%	0.08%
Exoskeleton Frozen	ASV 8	Comamonadaceae (Proteobacteria)	5/5	9.49%	1.06%
	ASV 16	*Verrucomicrobium* (Verrucomicrobia)	5/5	4.87%	0.76%
	ASV 24	*Methylobacter* (Gammaproteobacteria)	5/5	3.84%	0.35%
	ASV 12	*Sphaerotilus* (Betaproteobacteria)	5/5	3.73%	0.20%
	ASV 29	Comamonadaceae (Betaproteobacteria)	5/5	2.80%	0.57%
	ASV 35	*Aquabacterium* (Betaproteobacteria)	5/5	2.73%	0.19%

^a^ Frequency was determined from the number of individuals found with that ASV. ^b^ Relative abundance was determined from the total number of each ASV identified within each storage group (i.e., ethanol and frozen).

## Data Availability

Raw sequences are deposited in the NCBI Sequence Reads Archive under BioProject ID PRJNA797466.
